# SYBR Green-based Real-Time PCR targeting kinetoplast DNA can be used to discriminate between the main etiologic agents of Brazilian cutaneous and visceral leishmaniases

**DOI:** 10.1186/1756-3305-5-15

**Published:** 2012-01-12

**Authors:** Daniela Pita-Pereira, Rachel Lins, Marcia P Oliveira, Rosimar B Lima, Bernardo AS Pereira, Otacilio C Moreira, Reginaldo P Brazil, Constança Britto

**Affiliations:** 1 Laboratório de Biologia Molecular e Doenças Endêmicas, Instituto Oswaldo Cruz, FIOCRUZ, Pavilhão Leônidas Deane, sala 209, Avenida Brasil 4365, Manguinhos, 21040-360 Rio de Janeiro, RJ, Brazil; 2 Laboratório de Biologia Molecular de Insetos, Instituto Oswaldo Cruz, FIOCRUZ, Rio de Janeiro, RJ, Brazil; 3 Laboratório Interdisciplinar em Pesquisas Médicas, Instituto Oswaldo Cruz, FIOCRUZ, Rio de Janeiro, RJ, Brazil; 4 Laboratório de Bioquímica e Fisiologia de Insetos, Instituto Oswaldo Cruz, FIOCRUZ, Rio de Janeiro, RJ, Brazil

**Keywords:** SYBR Green Real-time PCR, Leishmaniases, kinetoplast DNA, thermal dissociation curves, molecular diagnosis, Brazil

## Abstract

**Background:**

Leishmaniases control has been hampered by the unavailability of rapid detection methods and the lack of suitable therapeutic and prophylactic measures. Accurate diagnosis, which can distinguish between *Leishmania *isolates, is essential for conducting appropriate prognosis, therapy and epidemiology. Molecular methods are currently being employed to detect *Leishmania *infection and categorize the parasites up to genus, complex or species level. Real-time PCR offers several advantages over traditional PCR, including faster processing time, higher sensitivity and decreased contamination risk.

**Results:**

A SYBR Green real-time PCR targeting the conserved region of kinetoplast DNA minicircles was able to differentiate between *Leishmania *subgenera. A panel of reference strains representing subgenera *Leishmania *and *Viannia *was evaluated by the derivative dissociation curve analyses of the amplified fragment. Distinct values for the average melting temperature were observed, being 78.95°C ± 0.01 and 77.36°C ± 0.02 for *Leishmania *and *Viannia*, respectively (p < 0.05). Using the Neighbor-Joining method and Kimura 2-parameters, the alignment of 12 sequences from the amplified conserved minicircles segment grouped together *L*. (*V*.) *braziliensis *and *L*. (*V*.) *shawii *with a bootstrap value of 100%; while for *L*. (*L*.) *infantum *and *L*. (*L*.) *amazonensis*, two groups were formed with bootstrap values of 100% and 62%, respectively. The lower dissociation temperature observed for the subgenus *Viannia *amplicons could be due to a lower proportion of guanine/cytosine sites (43.6%) when compared to species from subgenus *Leishmania *(average of 48.4%). The method was validated with 30 clinical specimens from visceral or cutaneous leishmaniases patients living in Brazil and also with DNA samples from naturally infected *Lutzomyia *spp. captured in two Brazilian localities.

**Conclusions:**

For all tested samples, a characteristic amplicon melting profile was evidenced for each *Leishmania *subgenus, corroborating the data from reference strains. Therefore, the analysis of thermal dissociation curves targeting the conserved kinetoplast DNA minicircles region is able to provide a rapid and reliable method to identify the main etiologic agents of cutaneous and visceral leishmaniases in endemic regions of Brazil.

## Background

The leishmaniases represent a group of diseases with worldwide distribution and a wide spectrum of clinical presentations, which constitute an important public health problem. Their control has been hampered by the unavailability of rapid means of detection and the lack of suitable therapeutic or prophylactic measures. In the Americas, members from the subgenus (*Viannia*), including *Leishmania *(*Viannia*) *braziliensis *and *Leishmania *(*Viannia*) *panamensis*, and from the *Leishmania *(*Leishmania*) *mexicana *complex cause the majority of cutaneous disease cases, whereas *Leishmania *(*Leishmania*) *infantum *(Syn. *L. chagasi*) is associated with visceral disease [1,2]. Cutaneous leishmaniasis (CL) in Brazil is caused by a variety of dermotropic *Leishmania *species and a great diversity of these parasites is found in the Amazon Region. Except in primary forest areas in North Brazil and the Amazon Region, *L*. (*V*.) *braziliensis *is the main widespread etiologic agent of CL in Brazil [3].

An accurate diagnostic method that allows the distinction between *Leishmania *isolates with overlapping geographic distribution is necessary to enable appropriate prognosis, epidemiology and therapy conducts. To this aim, molecular methods have been increasingly employed in an effort to detect infection and categorize *Leishmania *parasites up to genus, complex or species level [4]. Epidemiological data (clinical presentation and area of endemicity etc) are therefore of prime interest to complement molecular diagnosis. Different PCR-based methods targeting microsatellites, kinetoplastic DNA (kDNA), telomeric sequences, or gp63, hsp70, miniexon, β-tubulin, and rRNA genes have already been proposed [5]. More recently, real-time PCR, a platform that can process a sample in less than an hour, has been reported to rapidly differentiate even single nucleotide mutations within a target DNA sequence [6-13]. These assays are able to distinguish between groups of *Leishmania*, such as parasites from the *L*. (*L*.) *donovani *complex, and from the subgenus *(Viannia) *and other species from the subgenus *(Leishmania) *[10,12]. With the use of TaqMan probes directed to the *Leishmania *glucose-6-phosphate dehydrogenase locus, it was possible to differentiate *L*. (*V*.) *braziliensis *from other *Viannia *species and from those of subgenus *Leishmania *[7].

The aim of the present study was to evaluate the potential use of the conserved motif of kDNA minicircles in a SYBR Green-based real-time PCR approach, to discriminate between *Leishmania *subgenera and, therefore identify the main etiologic agents of cutaneous (CL) and visceral (VL) leishmaniases in Brazil, by exploiting differences in the kinetic dissociation profiles of the amplified DNA. The method was successfully applied to identify *Leishmania *species in 30 clinical samples collected from Brazilian patients with confirmed diagnosis of cutaneous or visceral disease, and also to confirm the infection by *L*. (*V*.) *braziliensis *or *L*. (*L*.) *infantum *in wild sand flies captured in different localities of the country.

## Methods

### Organisms, cell culture and DNA extraction

Promastigotes of *Leishmania (V.) braziliensis *(MHOM/BR/1975/M2903), *L. (V.) shawi *(MCEB/BR/1984/M8408), *L*. (*V*.) *guyanensis *(MHOM/BR/1975/M4147), *L*. (*V*.) *lainsoni *(MHOM/BR/1981/M6426), *L*. (*V*.) *naiffi *(MDAS/BR/1979/M5533), *L. (L.) infantum (Syn. L. chagasi) *[2] (MHOM/BR/1974/PP75) and *L. (L.) amazonensis *(MHOM/BR/1977/LTB0016) were provided by the *Leishmania *collection from Fiocruz (CLIOC), Rio de Janeiro. Epimastigotes from *Trypanosoma cruzi *(Cl-Brener and DM28c strains), *Endotrypanum monterogei, Herpetomonas muscarum muscarum *(ATCC 30260), *Phytomonas sp*. and *Crithidia fasciculata *(ATCC11745), were obtained from the Protozoa collection from Fiocruz (COLPROT), Rio de Janeiro. Parasites were cultivated in 3.7% brain heart infusion (BHI) medium supplemented with 10% heat-inactivated fetal bovine serum, penicillin 500 U/mL and streptomycin 500 mg/mL at 28°C for 4 days to reach late-log phase growth. Cells were harvested, washed and resuspended in phosphate-buffered saline (PBS) pH 7.4. Cell pellets containing 300 μL PBS were incubated with proteinase K (at a final concentration of 300 μg/mL) at 55°C for 2 h, and DNA was extracted by the sodium dodecyl sulfate/phenol extraction method [14]. The extracted DNA was precipitated with ethanol, dissolved in 50 μL of TE buffer (10 mM Tris-HCl, 1 mM EDTA) and quantified by measuring absorbance at 260 nm.

### Clinical specimens

5 mM EDTA-supplemented peripheral blood and bone marrow aspirates, as well as tissue biopsy specimens stored in 0.9% sodium chloride solution, were obtained from 30 patients with confirmed leishmaniasis, as follows. Fifteen of these patients were children considered to be VL carriers, living in the municipality of Campo Grande (Mato Grosso do Sul State) with ages ranging from 2-13 years old, and presenting characteristic clinical features (fever, hepatosplenomegaly and pancytopenia) associated to the detection of parasites in bone marrow aspirates by means of direct microscopy or in NNN (Novy-MacNeal-Nicolle) culture, and/or positive serology (≥ 1:80) as determined by indirect immunofluorescence (IIF). The remaining fifteen patients were from the municipality of Rio de Janeiro (Rio de Janeiro State), inhabiting areas with well-known occurrences of CL related to *L*. (*V*.) *braziliensis *and previously diagnosed by Giemsa staining of biopsy smears, histopathological examination of biopsies, or culture and/or PCR-RFLP on DNA extracted from lesion biopsies [15]. Samples corresponding to 1 mm^3 ^of skin biopsies obtained from the borders of the lesions, as well as 300 μL of EDTA-supplemented peripheral blood or bone marrow aspirates were used for DNA isolation, according to previously described protocols [15,16]. All DNA samples from clinical specimens were kindly provided by the National Reference Laboratory in Molecular Diagnosis of Leishmaniases, Instituto Oswaldo Cruz/Fiocruz, Rio de Janeiro (approved by the Ethics Committee of Fiocruz - protocol No. 503/09).

### *Lutzomyia *sand fly samples

Wild sand flies positive for infection with either *L*. (*V*.) *braziliensis *or *L*. (*L*.) *infantum *were divided into pools (10 insects per pool). DNA extraction and multiplex PCR assays followed by isotopic hybridization with specific *Leishmania *probes were performed as previously described [17,18]. A total of eleven positive *Lutzomyia *female pools were evaluated, consisting of: (*i*) five *Lu. intermedia *pools and three *Lu. migonei *pools collected in 2003, in two areas from the municipality of Rio de Janeiro (Rio de Janeiro State) with notification of CL [17]; (*ii*) two *Lu. cruzi *and one *Lu. forattinii *pools collected in an endemic area of VL in the municipality of Corumbá (Mato Grosso do Sul State), in May/June 2006 [18].

### Real-time PCR assays

SYBR Green-based real-time PCR was performed with primers directed to the conserved motif of *Leishmania *kinetoplast DNA minicircles [5'-GGC CCA CTA TAT TAC ACC AAC CCC-3' and 5'-GGG GTA GGG GCG TTC TGC GAA-3'] [19]. The reaction mixture contained 1× Power SYBR Green Master Mix (Applied Biosystems, Foster City, CA, USA), 1 pmol of each primer, 25 ng of template DNA and distilled ultra-pure water for a final reaction volume of 15 μL. The reactions were set up, in triplicate, in a 96-well optical reaction plate in an ABI Prism 7500 Sequence Detection System (Applied Biosystems, Foster City, CA, USA). The PCR conditions were as follows: an initial 12-min incubation step at 94°C, followed by 35 cycles of 30 s at 94°C, 30 s at 55°C, and 30 s at 72°C. The generation of amplification plots and dissociation kinetic analyses were according to the manufacturer's protocol. After amplification, the melting curve test was performed with an initial denaturation step at 95°C for 5 s, followed by 15 seconds at 50°C and continuous heating at 0.1°C/s to 90°C. Calculation of the melting temperature for each amplicon (*Tm*) was done directly by the equipment software. Each assay was repeated at least 3 times to check the reproducibility and reliability. Stringent measures to control sample contamination included two non template negative controls (NTC - reaction mix without DNA and distilled water alone) and DNA obtained from healthy donors' peripheral blood or from uninfected *Lutzomyia *(male specimen), in each reaction plate. Positive controls (50 ng *Leishmania *DNA) were also included.

### Conventional PCR for cloning

DNA samples from four *Leishmania *reference strains - *L*. (*V*.) *braziliensis, L*. (*V*.) *shawi, L*. (*L*.) *infantum *and *L*. (*L*.) *amazonensis*, were submitted to a conventional PCR using the same primer pair described for the real-time PCR. The 50 μL reaction consisted of 5 μL DNA template, 5 pmol primers, 1× Taq polymerase buffer, 1.25 U Taq DNA polymerase (Applied Biosystems, Foster City, CA, USA), 4.5 mM MgCl2, 100 μM of each dNTP (Invitrogen Corporation, Carlsbad, CA, USA) and distilled ultra-pure water. The mixture was incubated in a Perkin-Elmer thermocycler (GeneAmp PCR System 9600; Applied Biosystems, Foster City, CA, USA), following the same amplification conditions reported for the real-time PCR. Ten microlitres of amplification product were resolved in 2% agarose gel electrophoresis, stained with GelRed^® ^(Sigma-Aldrich, St. Louis, MO, USA) and visualized under UV-light. The remaining 40 μL were purified using the commercial kit Wizard ^® ^SV Gel and PCR Clean-up System (Promega, Madison, WI, USA), according to the manufacturer's protocol. After DNA quantification by spectrophotometry, the fragments were cloned using TOPO TA cloning kit ^® ^(Invitrogen Corporation, Carlsbad, CA, USA) following the manufacturer's recommendation.

### Sequence Analysis

For each *Leishmania *reference strain, three clones derived from the amplified conserved minicircles region were sequenced using the BigDye Terminator Cycle Sequencing Kit Ready Reaction version 3.1 on the ABI Prism 3100 Avant Genetic Analyzer (Perkin-Elmer, Applied Biosystems, Foster City, CA, USA), following the manufacturer's specifications. Sequence homology searches were made by the online NCBI BLAST software http://blast.ncbi.nlm.nih.gov/Blast.cgi. Sequences were aligned using the CLUSTAL software in the BioEdit software package [20]. A tree was constructed using the MEGA 3.1 software [21], applying the Neighbor-Joining method and Kimura 2-parameters. Stability of the tree was confirmed by bootstrap analysis.

## Results and discussion

### Limited nucleotide variation of the kDNA conserved motifs promote distinctive melting profiles for *Leishmania *subgenera

**Figure 1:**
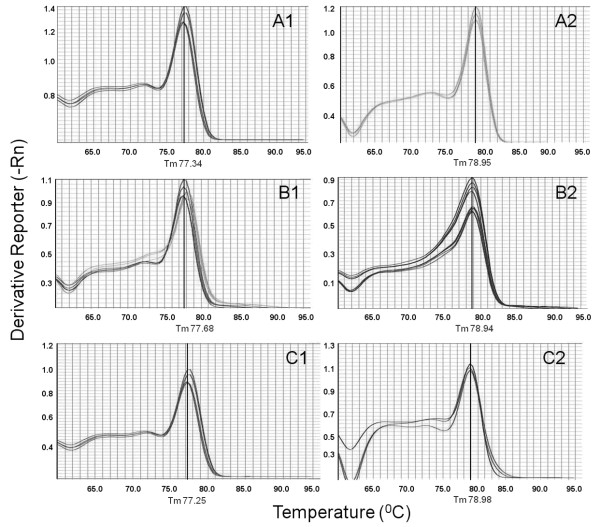
**Melting curve analyses of kDNA conserved regions amplicons**. Representative SYBR Green dissociation curves of kDNA amplified products to determinate the melting temperature values [Tm]. Following amplification in the ABI Prism 7500 Sequence Detection System, the amplicons were submitted to a gradual temperature increase (0.1°C/s to 90°C). The Tm calculation of each amplicon was done directly by the software provided (indicated by the perpendicular line in the graphics). Each trace in the charts represents a single analyzed DNA sample. The graphics on the left represent typical kinetic dissociation profiles obtained for the *Viannia *subgenus: (A1) *L. (V.) shawi *and *L. (V.) braziliensis *promastigote reference strains; (B1) clinical samples from cutaneous disease patients; (C1) *Lu. intermedia and Lu. migonei *pools naturally infected by *L. (V.) braziliensis*. On the right are represented the melting profiles for the subgenus *Leishmania*: (A2) *L. (L.) amazonensis *and *L. (L.) infantum *promastigote reference strains; (B2) clinical specimens from visceral disease patients; (C2) *Lu. cruzi *and *Lu. forattinii *pools naturally infected by *L*. (*L*.) *infantum*.

Genomic DNA extracted from four *Leishmania *promastigote reference strains, representing both *Viannia *and *Leishmania *subgenera, was assayed. For each subgenus, the SYBR Green melting curve analysis showed a single peak indicating its specificity, which was confirmed by fractionation in an agarose gel. Figure 1 demonstrates the typical dissociation curves representing specific melting temperatures (Tm) generated by the kDNA amplified products of promastigote reference strains from *Viannia *and *Leishmania *subgenera [Figure 1- A1 and A2, respectively]. *Leishmania *(*L*.) *infantum *and *L*. (*L*.) *amazonensis*, both from subgenus *Leishmania*, presented an average Tm of 78.95°C ± 0.01, and the two *Viannia *species, *L*. (*V*.) *braziliensis *and *L*. (*V*.) *shawi*, revealed a slightly lower but statistically distinct Tm value of 77.34°C ± 0.01 (p < 0.001 - Mann-Whitney Rank Sum Test).

**Figure 2:**
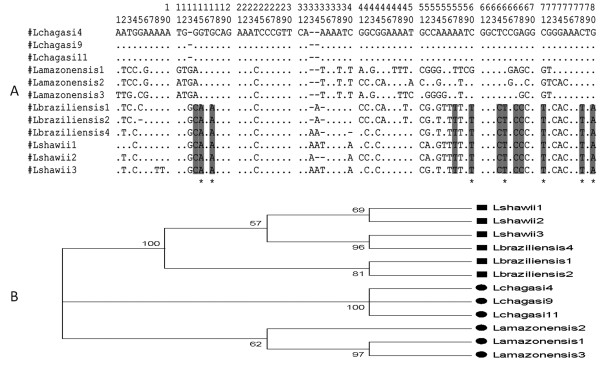
**Sequence alignment of amplified minicircles kDNA conserved region and phylogenetic analysis**. A - Alignment of 12 generated sequences representing three clones for each analyzed *Leishmania *reference strain. Highlighted in gray: Exclusively *L*. (*Viannia*) subgenus conserved nucleotides. Most of the substitutions shown are possibly associated with the lower melting temperature (Tm) characteristic for this subgenus (indicated by asterisks). B - Neighbor-Joining tree based on kDNA amplicons sequence. The rectangles represent sequences from subgenus *Viannia *and circles indicate those from subgenus *Leishmania*. Bootstraps values are indicated.

Considering an estimated length for the *Leishmania *minicircles conserved region of around 120 base pairs, we further investigated these minor differences on the melting temperature for each subgenus by sequencing the amplification products obtained from the analyzed *Leishmania *species. A kDNA-based conventional PCR was performed and the amplified 120 bp fragments were cloned. Three clones were selected for sequencing for each *Leishmania *species and 12 generated sequences were aligned, revealing slight variations (nucleotide substitutions, insertions and/or deletions). All 12 sequences are shown in an additional file [see Additional file 1]. Despite the limited number of nucleotide variations in the *Leishmania *kDNA conserved motif, it was possible to construct a tree based on the Neighbor-Joining method and Kimura 2-parameters [Figure 2]. We observed the formation of a very distinct group with a bootstrap value of 100% comprising *L*. (*V*.) *shawi *and *L*. (*V*.) *braziliensis*, from the subgenus *Viannia*. This group revealed in its sequence composition a lower G/C content (43.6%) when compared to reference strains from the subgenus *Leishmania*: *L*. (*L*.) *infantum *(48.1%) and *L*. (*L*.) *amazonensis *(48.7%). Taking into consideration that the kDNA amplified fragments were of similar lengths, this difference in G/C ratio could explain the lower dissociation temperature observed for DNA amplicons derived from subgenus *Viannia *(77.34°C ± 0.01). For the *Leishmania *subgenus, both analyzed species formed groups with significant bootstrap values - 100% for *L*. (*L*.) *infantum *and 62% for *L*. (*L*.) *amazonensis *[Figure 2]. Differences in base composition between these two species did not interfere with the dissociation curve kinetics calculated for the respective amplified products.

In order to verify if other *Viannia *species causing CL in Brazil generated the characteristic melting profiles for the kDNA amplicons previously observed for this subgenus, the panel of *Viannia *reference strains was expanded by including *L*. (*V*.) *guyanensis, L*. (*V*.) *naiffi *and *L*. (*V*.) *lainsoni *promastigotes [see Additional file 2]. No significant differences were observed between the Tm values for these species, with an average of 77.36°C ± 0.02 (p > 0.05). Table 1 presents the Tm values generated from the dissociation curve analysis for each tested *Leishmania *reference strain from both subgenera. Even though different species from the same subgenus presented similar melting points, the melting curve analyses were able to distinguish between the two main etiologic agents of cutaneous and visceral leishmaniases in Brazil, *L*. (*V*.) *braziliensis *and *L*. (*L*.) *infantum*, respectively.

**Table 1:** Differentiation between *Leishmania *subgenera by SYBR Green melting temperatures (Tm) from amplified minicircles kDNA conserved region

Source	Samples	Origin	Melting Temperature (°C ± SD)
Promastigote reference strains	*L (V.) braziliensis *(MHOM/BR/1975/M2903)	IOC/FIOCRUZ	77.34 ± 0.01
	
	*L (V.) shawi *(MCEB/BR/1984/M8408)	IOC/FIOCRUZ	77.34 ± 0.01
	
	*L (V.) naiffi *(MDAS/BR/1979/M5533)	IOC/FIOCRUZ	77.34 ± 0.01
	
	*L (V.) lainsoni *(MHOM/BR/1981/M6426)	IOC/FIOCRUZ	77.37 ± 0.01
	
	*L (V.) guyanensis *(MHOM/BR/1975/M4147)	IOC/FIOCRUZ	77.39 ± 0.01
	
	*L (L.) infantum (Syn. L. chagasi) *(MHOM/BR/1974/PP75)	IOC/FIOCRUZ	78.95 ± 0.01
	
	*L (L.) amazonensis *(MHOM/BR/1977/LTB0016)	IOC/FIOCRUZ	78.94 ± 0.00

Human clinical specimens	Cutaneous leishmaniasis (skin biopsies)	Rio de Janeiro/RJ	77.68 ± 0.38
	
	Visceral leishmaniasis (peripheral blood and bone marrow aspirates)	Campo Grande/MS	78.94 ± 0.37

Field sand flies	*Lutzomyia intermedia*	Rio de Janeiro/RJ	77.25 ± 0.15
	
	*Lutzomyia migonei*	Rio de Janeiro/RJ	77.25 ± 0.15
	
	*Lutzomyia cruzi*	Corumbá/MS	78.98 ± 0.10
	
	*Lutzomyia forattini*	Corumbá/MS	78.98 ± 0.10

IOC: Oswaldo Cruz Institute, FIOCRUZ: Oswaldo Cruz Foundation, RJ: State of Rio de Janeiro, MS: State of Mato Grosso do Sul, SD: standard deviation

The melting temperatures of kDNA amplified products were analyzed from samples obtained from *Leishmania *promastigotes reference strains, clinical specimens from cutaneous and visceral leishmaniases patients and field-captured phlebotomine insects. The dissociation curves analyses revealed distinct melting temperatures between the subgenera *Viannia *and *Leishmania *(p < 0.001 - Mann-Whitney Rank Sum Test).

### Methodology validation with human clinical specimens and wild sand flies

The findings revealed through the analyses of *Leishmania *promastigote reference strains were corroborated with the inclusion of DNA samples obtained from clinical specimens (peripheral blood, bone marrow aspirates and skin biopsies) of patients living in Brazilian leishmaniasis endemic areas and with confirmed diagnosis of visceral or cutaneous disease. To ensure the reproducibility of the assays, positive controls - DNA from *L*. (*V*.) *braziliensis, L*. (*V*.) *shawi, L*. (*L*.) *infantum *and *L*. (*L*.) *amazonensis *reference strains, were included in each run. Figure 1 shows the characteristic kinetic dissociation profiles for the kDNA amplicons derived from human clinical samples. The results with the VL samples indicated a typical Tm value for the subgenus *Leishmania *(78.94°C ± 0.37) [Figure 1, B2], which is differentiated from the Tm observed for the analyzed dermotropic clinical specimens (77.68°C ± 0.38) [Figure 1, B1]. These data suggest that the technique of dissociation curve analysis has the potential to be applied to discriminate between *L*. (*V*.) *braziliensis *and *L*. (*L*.) *amazonensis *infection in areas of Brazil where the distribution of these species overlaps. As for cases of visceral disease in Brazilian patients, this methodology is able to identify the etiologic agent *L*. (*L*.) *infantum*. The specificity of the method was confirmed with DNA extracted from healthy donors peripheral blood.

The molecular method was also able to differentiate between natural infections caused by *L*. (*V*.) *braziliensis *or *L*. (*L*.) *infantum *in *Lutzomyia *sand flies, which were previously positive in a diagnostic assay by multiplex conventional PCR following hybridization [17,18]. The assays were performed with DNA samples obtained from five pools of *Lu. intermedia *and three pools of *Lu. migonei *(10 insects/pool) collected in the municipality of Rio de Janeiro, in areas with notification of CL in human and dogs [17]; and, also, DNA extracted from two pools of *Lu. cruzi *and one pool of *Lu. forattinii *from specimens captured in an endemic VL area in the municipality of Corumbá, Mato Grosso do Sul State [18]. As displayed in Figure 1 the data from these specimens were in accordance with our findings, revealing kinetic dissociation profiles compatible with the characteristic Tm previously observed for *L*. (*V*.) *braziliensis *(77.25°C ± 0.15) in pools of *Lu. intermedia/Lu. migonei *[Figure 1, C1], and for *L*. (*L*.) *infantum *(78.98°C ± 0.10) in pools of *Lu. cruzi/Lu. forattinii *[Figure 1, C2].

DNAs from other Trypanosomatids that also parasitize the hosts of *Leishmania*, such as *Trypanosoma cruzi *(mammalian host) and *Endotrypanum *(invertebrate host), as well as other species closely related (*Herpetomonas, Phytomonas, Crithidia*) were tested for the specificity of the assay. DNA from uninfected *Lutzomyia *was also assayed as a negative control. There was no amplification of DNA in these assays.

Table 1 summarizes the data obtained through the analyses of cultivated *Leishmania *promastigotes, human clinical specimens and naturally infected phlebotomine sand flies.

### Molecular markers and PCR-based systems for the diagnosis of *Leishmania *infection

Restriction fragment length polymorphism (RFLP) analyses of PCR-amplified products from multicopy genes have shown promising results in detecting *Leishmania *species and in clarifying the molecular diversity and relationships within *Leishmania *spp. [22-24]. PCR-based methods with further molecular typing by sequence analysis have also been described [25,26]. Real-time PCR is currently considered as an emerging technology for the detection, genetic characterization and quantification of protozoan parasites. By using the Light-Cycler SYBR Green system targeting minicircles kDNA, Nicolas et al. (2002) were able to differentiate four important *Leishmania *species from the Old World [8]. More recently, the performance of PCR-RFLP for the internal transcribed spacer of ribosomal RNA (ITS1) and SYBR Green-based real-time PCR focused on kDNA were compared, with the aim of identifying *L*. (*L*.) *infantum, L*. (*L*.) *major *and *L*. (*L*.) *tropica/killicki*, the etiologic agents of CL in Tunis, directly from human skin scrapings [6]. This comparison revealed a failure of the kDNA real-time PCR method in identifying the Tunisian *L*. (*L*.) *tropica/L*. (*L*.) *killicki *in 5 out of 27 samples, which the authors attributed to the kinetoplast DNA polymorphism found in *Leishmania *strains. Thus, it was suggested that standardization of kDNA real-time PCR is needed to allow interlaboratory comparisons and maximize repeatability. A recent investigation reported the discriminatory power of a new high-resolution tool for the dissociation analysis of PCR products derived from the ITS1 gene [11]. The high resolution melt analysis (HRM) could distinguish between all Old World *Leishmania *species causing human disease, except *L*. (*L*.) *infantum *from *L*. (*L*.) *donovani*, which presented similar HRM curves. Other studies also demonstrated the ability of melting curve analysis to distinguish *Leishmania *parasites up to subgenus, complex or species level, through the use of different targets, such as the 18S rDNA sequences [10], gp63 [27] and glucose-6-phosphate dehydrogenase (g6pd) [7]. Alternatively, a TaqMan-based real-time PCR for the detection of glucosephosphate isomerase (GPI) gene was able to discriminate between four *Leishmania *groups - subgenus *Viannia *and the complexes *L. (L.) mexicana, L. (L.) donovani/infantum *and *L. (L.) major *[12].

Accurate and sensitive procedures for the diagnosis of *Leishmania *infection and species identification directly from clinical material or through the analysis of phlebotomine sand flies are still required to enable adequate treatment and appropriate leishmaniases control. Identification of the prevalent *Leishmania *species in sand flies can alert clinicians to potential subsequent human cases in a determined geographic area. In this sense, the use of kDNA minicircles on PCR-based assays coupled to isotopic hybridization has been shown to offer enhanced sensitivity over more traditional diagnostic methods to evaluate natural infection in sand fly vectors from Brazil, allowing DNA detection corresponding to only one *Leishmania *parasite presented in pools of 10 male phlebotomine insects in reconstituted samples [17]. This high sensitivity is partly due to the elevated copy number of the target kDNA minicircles, which are represented in approximately 10,000 copies per parasite genome. Compared with conventional PCR tests targeting kinetoplast DNA, the most important implication of the assay herein reported is the ability to rapidly discriminate among the pathogenic species representing the most common causative agents associated to CL or VL in Brazil, thus optimizing the identification of *Leishmania *DNA in sand flies or clinical specimens without the need of posterior detection of amplicons by gel electrophoresis or hybridization step.

PCR-based assays directed to kDNA are currently the most sensitive methodology for the diagnosis of either visceral or cutaneous leishmaniasis [28]. The samples tested in the present study were selected, as they were positive by traditional diagnostic methods (microscopy/culture and/or serology, Giemsa staining/histopathology), as well as PCR, before being subjected to testing with this assay. In all cases, the SYBR Green melting curve profile was able to corroborate the predetermined diagnosis. As formerly reported, the existence of shifted and overlapping *Tm *values indicates kinetoplast DNA polymorphism among *Leishmania *strains, which causes a variation of the amplified sequence and, consequently, of the corresponding melting temperature [6]. Nevertheless, the kDNA polymorphism observed in the present study is too limited to allow the use of SYBR Green-based real-time PCR as an intraspecific typing method. Other methods, such as the analysis of microsatellite markers, are more appropriate for this specific issue [29].

## Conclusions

Based on the sequence-dependent thermal dissociation properties, despite the analysis of a relatively small sample set, our data suggest that SYBR Green-based real-time PCR targeting the conserved minicircles region from kinetoplast DNA provides a rapid, sensitive and simple alternative for the precise identification of *Leishmania *subgenera. This methodology was able to discriminate among the human-pathogenic species representing the most common causative agents of cutaneous and visceral leishmaniases in Brazilian endemic areas. Similar kinetic dissociation profiles were observed for the amplified products derived from subgenus *Viannia *reference strains, skin biopsies from *L. (V.) braziliensis*-related CL patients and *Lutzomyia *sand flies infected by *L*. (*V*.) *braziliensis*. Likewise, the analyses performed with promastigotes from subgenus *Leishmania *were in accordance to the results obtained with the set of clinical specimens from VL patients and sand fly vectors infected by *L*. (*L*.) *infantum*.

## List of abbreviations

kDNA: kinetoplast DNA; SYBR Green: N', N'-dimethyl-N-[4-[(E)-(3-methyl-1,3-benzothiazol-2-ylidene) methyl]-1-phenylquinolin-1-ium-2-yl]-N-propylpropane-1,3-diamine; PCR: Polymerase Chain Reaction; Tm: melting temperature; CL: Cutaneus Leishmaniasis; VL: Visceral Leishmaniasis; gp63: major surface glycoprotein of 63-kDa; hsp70: heat shock protein of 70-kDa; rRNA: ribosomal RNA; G/C: guanine/cytosine; RFLP: Restriction fragment length polymorphism; ITS1: Internal Transcribed Spacer; 18S rDNA: small subunit of the ribosomal DNA; EDTA: Ethylenediamine tetraacetic acid.

## Competing interests

The authors declare that they have no competing interests.

## Authors' contributions

DPP designed the experiments, carried out the molecular studies (PCR-based assays and sequencing), participated in the sequence alignment, and helped to draft the manuscript. RL participated with the sequence alignment and with the construction and interpretation of the tree. MPO and RBL provided the DNA samples from human clinical specimens, and were responsible for performing all previous diagnostic tests for the CL and VL patients included in this study. BASP and OCM participated with the statistical analysis, adaptation of figures and table, and revising the English grammar. RPB contributed to sand fly captures and the taxonomic identification of *Lutzomyia *specimens. CB conceived and designed the study, and wrote the final version of the manuscript. All authors read and approved the final manuscript.

## Supplementary Material

Additional file 1 **Kinetoplast DNA minicircles conserved region sequences**. This file contains the 12 generated sequences from amplified kDNA minicircles conserved region, representing three clones for each analyzed *Leishmania *reference strain [*L*. (*V*.) *braziliensis, L*. (*V*.) *shawi, L*. (*L*.) *infantum chagasi, L*. (*L*.) *amazonensis*]. Click here for file

Additional file 2 **Melting curve analyses of the conserved motifs of kDNA amplicons**. This file displays the characteristic SYBR Green dissociation profiles of kDNA amplified conserved regions after submitting the amplicons to a gradual temperature increase. The upper graphic represents reference strains of the *Leishmania *subgenus - *L. amazonensis, L. infantum*, with an estimated melting temperature (Tm) of 78.95°C ± 0.01. The lower graphic shows the resulting melting analysis for the *Viannia *subgenus reference strains - *L. guyanensis, L. lainsoni, L. naiffi, L. braziliensis, L. shawi*, where an average Tm of 77.36°C ± 0.02 was found. There was a significant difference between the Tm values of the two *Leishmania *subgenera (p < 0.001 - Mann-Whitney Rank Sum Test). Click here for file
