# Supplementary figures and images for: SYBR Green-based Real-Time PCR targeting kinetoplast DNA can be used to discriminate between the main etiologic agents of Brazilian cutaneous and visceral leishmaniases
Source: Parasit Vectors. 2012 Jan 12;5:15. doi: 10.1186/1756-3305-5-15 (PMC3274473; doi:10.1186/1756-3305-5-15)

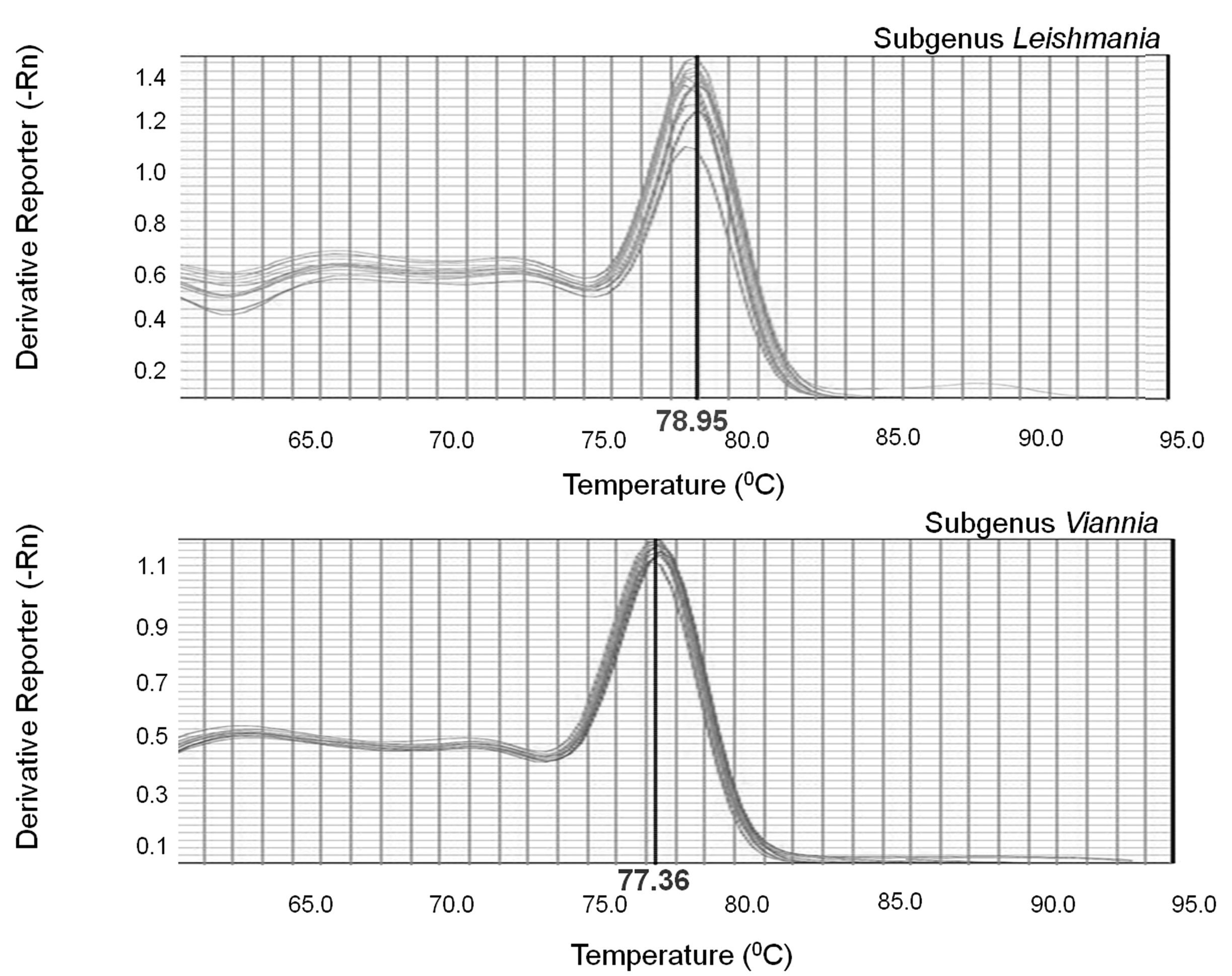

Supplement: Additional file 2 — Melting curve analyses of the conserved motifs of kDNA amplicons. This file displays the characteristic SYBR Green dissociation profiles of kDNA amplified conserved regions after submitting the amplicons to a gradual temperature increase. The upper graphic represents reference strains of the Leishmania subgenus - L. amazonensis, L. infantum, with an estimated melting temperature (Tm) of 78.95°C ± 0.01. The lower graphic shows the resulting melting analysis for the Viannia subgenus reference strains - L. guyanensis, L. lainsoni, L. naiffi, L. braziliensis, L. shawi, where an average Tm of 77.36°C ± 0.02 was found. There was a significant difference between the Tm values of the two Leishmania subgenera (p < 0.001 - Mann-Whitney Rank Sum Test). [file 1756-3305-5-15-S2.TIFF]
